# Twists and Turns: Gallbladder Volvulus in an Elderly Patient

**DOI:** 10.7759/cureus.65661

**Published:** 2024-07-29

**Authors:** James C Zillman, Sandres Aodish, Tyler C Dobratz, Patrick Swaney, John Stivers

**Affiliations:** 1 School of Medicine, Lake Erie College of Osteopathic Medicine, Erie, USA; 2 Surgery, Lake Erie College of Osteopathic Medicine, Erie, USA; 3 Orthopedic Surgery, Lake Erie College of Osteopathic Medicine, Erie, USA; 4 General Surgery, University of Pittsburgh Medical Center, Erie, USA

**Keywords:** severe acute cholecystitis, right upper quadrant abdominal pain, geriatric patient, rare differential, diagnosis difficulty, laparoscopic cholecystectomy, necrotizing cholecystitis, surgical case report, gallbladder volvulus

## Abstract

Gallbladder volvulus (GBV) is a rare medical condition characterized by twisting of the gallbladder around its mesentery. The condition presents with a higher prevalence in older, thin, elderly women and is a challenging diagnosis with nonspecific symptoms often overlapping with acute cholecystitis. Early diagnosis and intervention are critical to prevent complications including ischemia, necrosis, gangrene, perforation, or sepsis. This case is about a 94-year-old woman who presented with epigastric and right upper quadrant pain, nausea, and vomiting with non-specific laboratory results and radiographic findings, leading to an intraoperative diagnosis of GBV. This report underscores the importance of considering GBV in differentials for acute abdominal signs and symptoms and the challenges in diagnosing GBV preoperatively due to its non-specific presentation and, in this case, unrevealing laboratory findings.

## Introduction

Gallbladder volvulus (GBV) is a rare condition accounting for 1 out of 365,520 hospital admissions [1,2]. The condition was first reported by Wendel in 1898 and described as a “floating gallbladder” [3]. Since then, approximately 500 occurrences have been documented in English literature [4,5]. The condition predominantly affects older women, and many patients identified with this condition are thin and have low amounts of visceral body fat, which often coincides with the aging process [1-3,6]. GBV occurs due to the rotation of the gallbladder on its axis along the cystic duct [5,6]. The rotation of the gallbladder can be described as either complete (>180 degrees of rotation) or incomplete (≤180 degrees of rotation) [6,7]. Symptoms vary depending on the extent of the rotation; patients with incomplete rotation typically exhibit biliary colic-like symptoms, while those with complete rotation present with signs similar to acute cholecystitis [8].

Given that the signs and symptoms of GBV commonly overlap with acute cholecystitis, this can cause a significant delay in identifying GBV. Further, GBV requires emergent detorsion and cholecystectomy, whereas acute cholecystitis may not require emergent surgical intervention. Thus, delay in surgical intervention may lead to an increased risk of complications [5]. These complications include ischemia, necrosis, gangrene, perforation, or sepsis [5-7]. Even with the high risk of complications from preoperative delay of diagnosis, only about 25 percent of diagnoses are made preoperatively [7]. Due to the myriad of complications, it is critical to diagnose GBV as early as possible so emergent cholecystectomy can be performed.

## Case presentation

A 94-year-old female presented to the emergency department with the abrupt onset of epigastric, chest, and right upper quadrant pain associated with nausea and vomiting. Her past medical history was significant for hypertension, coronary artery disease with ST-elevation myocardial infarction (STEMI), hyperlipidemia, and gastroesophageal reflux disease (GERD). Upon presentation, vitals demonstrated a blood pressure of 163/92, a pulse of 77, a respiratory rate of 20, a SaO2 of 97 on room air, and a temperature of 36.9 degrees Celsius.

The physical examination revealed the patient had a soft, non-distended abdomen with normoactive bowel sounds, right upper quadrant tenderness with a negative Murphy sign, and a palpable gallbladder. Laboratory studies were unrevealing with no leukocytosis, mildly elevated creatinine of 1.28, ALT/AST and lipase within normal limits, and negative high-sensitivity troponin. EKG obtained demonstrated sinus rhythm with first-degree AV block at a rate of 73 bpm, left axis deviation, and left bundle branch block, with no acute changes when compared to previous EKG. Computer tomography (CT) imaging of her abdomen and pelvis revealed a distended gallbladder demonstrating wall thickening and central biliary dilatation with the extrahepatic duct measuring up to 1.3 cm. Abdominal ultrasound revealed a dilated gallbladder with gallbladder wall thickening measuring 6 mm, no gallstones, and trace pericholecystic fluid. The common bile duct was mildly dilated measuring 9 mm but this is within normal limits for the patient's age and was similar in diameter to a previous CT.

**Figure 1:**
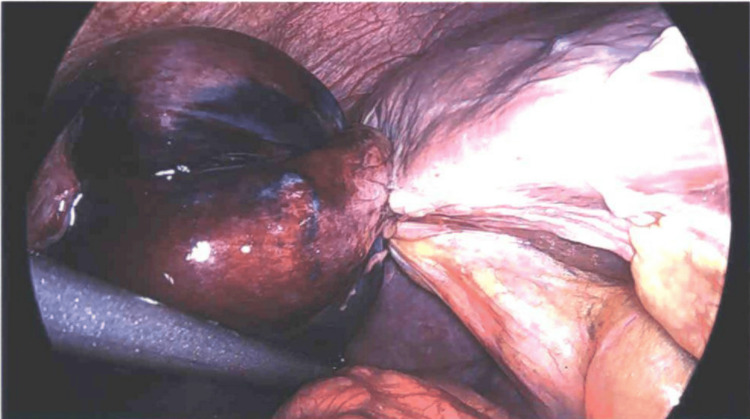
Gallbladder torsioned around the liver in a levorotational manner, stretching the cystic artery over the back of the attachment and bringing the lateral wall over the top and towards the camera view.

**Figure 2:**
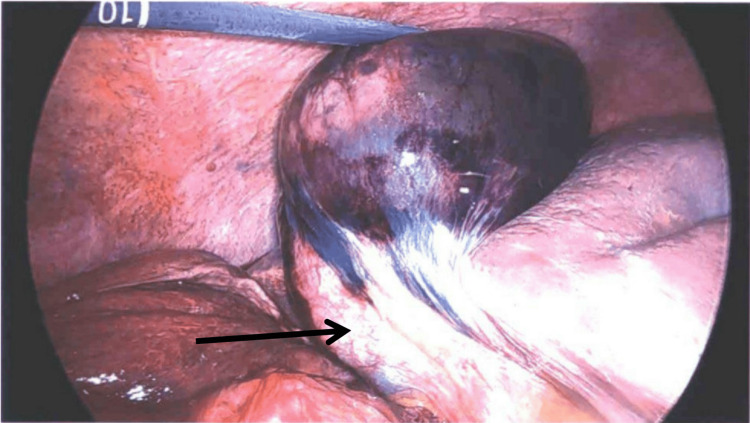
Following detorsion of the gallbladder with the cystic duct visible at the 7 o’clock position (black arrow).

**Figure 3:**
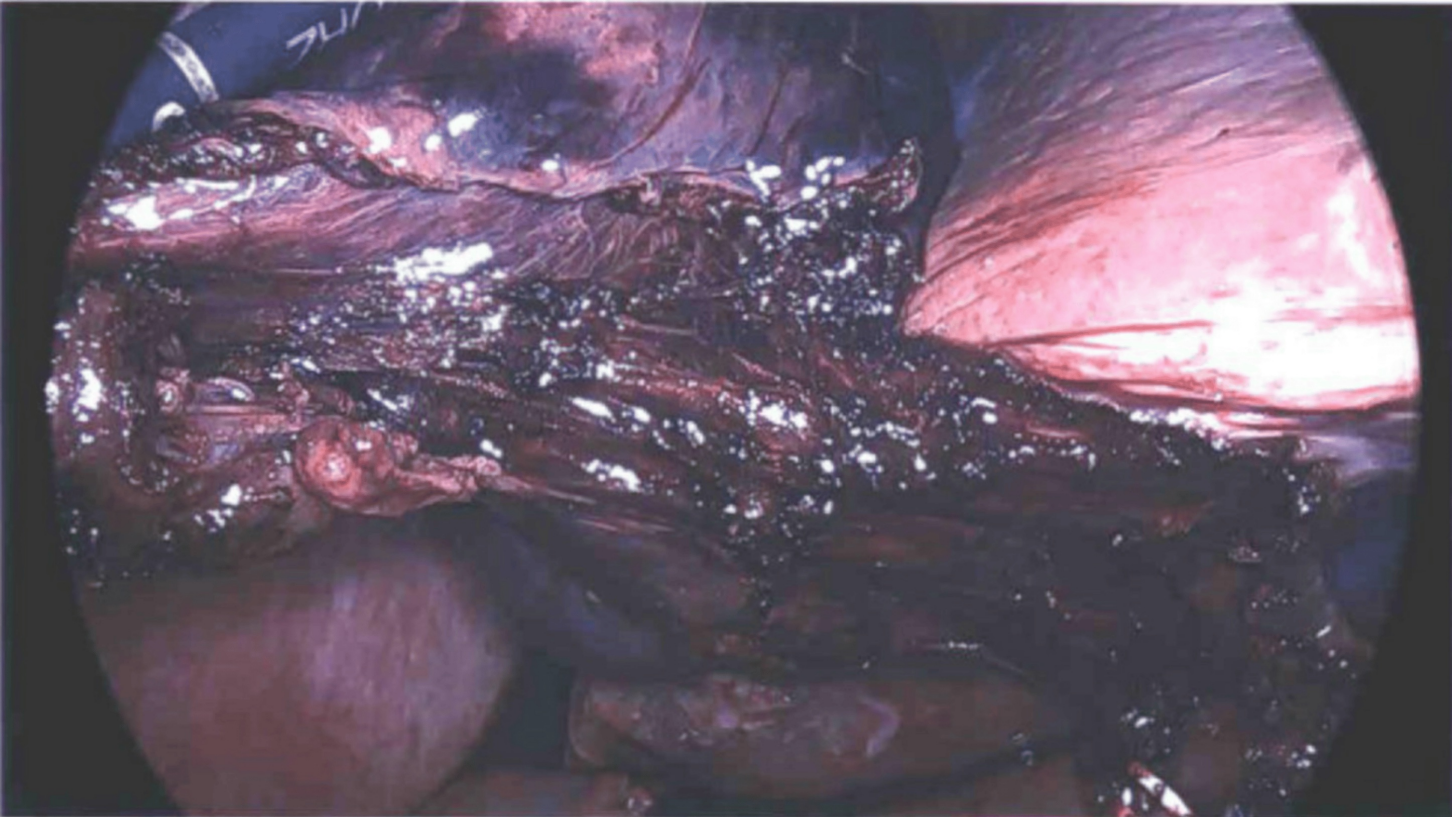
Following division of the cystic duct with metal clips visible at the 4 o’clock position demonstrating the length of attachment of the gallbladder to the liver after half of the liver attachments have been cauterized.

Due to her radiographic and clinical examination findings concerning acute cholecystitis, surgical consultation was obtained in the emergency department, and the patient was admitted to acute care surgery and scheduled for laparoscopic or possible open cholecystectomy. The patient was placed on N.P.O., started on IV cefoxitin, and consent was obtained for surgery that was performed the following morning. Intraoperatively, inspection of the gallbladder revealed a long mobile connection of the gallbladder to its fossa, and rotation of the gallbladder along this mesoaxial plane with apparent venous obstruction causing wall congestion and gangrene (Figures 1-3). Manual detorsion of the gallbladder was performed, and the cholecystectomy was completed without any complications. The patient was extubated and returned to the post-anesthesia care unit in stable condition. The patient had no post-operative complications and was discharged two days following surgery. The pathology report demonstrated acute necrotizing cholecystitis with interstitial hemorrhage and the absence of gallstones, supporting venous congestion as the torsion had obstructed the outflow into the gallbladder fossa.

## Discussion

GBV is a rare condition, sometimes described as “gallbladder torsion” and is characterized by the twisting of the gallbladder around its mesentery [1-5]. The pathology behind GBV is not fully understood, but multiple proposed etiologies exist. These include anatomical variants of the gallbladder such as abnormality of the vascular pedicle, as well as variants of the mesentery. The mesentery variants are Type A, a long and wide mesentery, and Type B, an incomplete or absent mesentery. Interestingly, gallstones do not need to be present and are not thought to contribute to GBV [1,5]. A long and mobile mesentery was seen in this patient.

Diagnosing a patient with GBV is challenging as the common signs and symptoms are nonspecific and can mimic acute infective pathologies, and there are no definitive laboratory or radiologic findings [1]. Patients commonly present with right upper quadrant abdominal pain, nausea, vomiting, fever, and a palpable abdominal mass [1,5,9]. Lab results often seen in GBV patients include leukocytosis, a raised C-reactive protein (CRP), and normal liver transaminases [1,4,8]. Hyperbilirubinemia is also considered a finding that can suggest GBV [9]. Diagnostic imaging, especially CT scans and ultrasounds, is vital in diagnosing GBV [1]. On ultrasound common features seen include detecting the GB outside its anatomical fossa, showing distension and linear echoes converging towards the tip of a “cone” [1,8]. On CT there are multiple criteria proposed by both Kitagawa et al. and Layton et al. [8]. Kitagawa et al. propose observing the following findings: the presence of fluid between the gallbladder and the gallbladder fossa, the horizontal lie of the gallbladder, the presence of an enhancing cystic duct situated on the gallbladder's right, and evidence of gallbladder inflammation or ischemia [10]. In contrast, Layton et al. suggest examining for gallbladder distension, the presence of a "beak and swirl sign" immediately distal to the fulcrum point, and a change in the anatomical position of the gallbladder from vertical to horizontal [11]. While it is a difficult diagnosis to make, it should be treated emergently with an open or laparoscopic cholecystectomy [1]. Failure to diagnose and treat rapidly can subject the patient to more serious complications such as infarction or necrosis of the gallbladder, bilious peritonitis, perforation, multiorgan failure, and death [1,5]. A delay in diagnosis by more than two days is also associated with increased mortality [5].

This case specifically highlights the challenges of diagnosing GBV preoperatively and the importance of considering GBV in the differential diagnosis for patients presenting with acute abdominal symptoms. As discussed above, this patient exhibited the typical patient characteristics: elderly, female, and thin body habitus. However, presenting symptoms did not fit the characteristics more indicative of GBV, with the patient lacking hyperbilirubinemia, fever, and leukocytosis [9]. CRP was also not obtained in this case. CT and ultrasound did reveal a distended and dilated gallbladder with wall thickening and pericholecystic fluid. The lack of many of the characteristic findings that can point a clinician towards GBV over acute cholecystitis further increased the difficulty of diagnosis [9].

One key feature of this case is the importance of physical exam findings in the preoperative diagnosis of GBV. Physical examination of the patient was positive for right upper quadrant tenderness and a palpable gallbladder, usually an ominous sign. In such a setting, surgeons should consider several possible causes of gallbladder inflammation including GBV, cholecystitis cholelithiasis, biliary dyskinesia, or acalculous cholecystitis. A well-performed physical examination can help expedite the time to operation and physical examinations must be addressed as part of the decision-making and diagnostic process.

Despite the initial evaluation not directly identifying GBV, due to the severe and acute presentation of symptoms causing concern for severe cholecystitis, the patient was admitted to acute care surgery and prepared for cholecystectomy. The GBV was discovered intraoperatively and successful detorsion with cholecystectomy was performed without complications. Even though this patient had a positive outcome, the preoperative presentation and findings could have warranted consideration of non-operative management of suspected cholecystitis, especially given no past medical history of recurrent cholecystitis. Again, this emphasizes the importance of considering GBV in a differential diagnosis for these symptoms. Failure to consider GBV and proceed to surgical intervention could be detrimental to the patient due to the potential progression of GBV as discussed above [1,5].

## Conclusions

In conclusion, we present a case of the uncommon condition of GBV in a 94-year-old woman after presenting to the emergency department with abrupt onset epigastric, chest, and right upper quadrant pain. Based on the presenting clinical symptoms and radiographic findings, the patient was suspected to have acute cholecystitis and was scheduled to have laparoscopic surgery the following morning. Intraoperative inspection revealed torsion of the gallbladder around its mesentery with gangrene necrosis of the gallbladder wall. Further delay in procedure or discovery of GBV could have resulted in worse complications and outcomes as evidenced by the formation of wall gangrene. The aforementioned emphasizes the need for an expedited preoperative diagnosis to prevent increased patient mortality and complications. Currently, there is no reliable method to diagnose GBV other than utilizing a combination of diagnostic tools and having a high clinical suspicion, which is complicated due to the non-specificity of symptoms. Diagnostic imaging is still the most crucial method to diagnose GBV but may not demonstrate abnormalities for definite confirmation of diagnosis. This case demonstrates the need for an early preoperative suspicion of GBV to prevent increased patient morbidity and mortality related to the condition.
